# Sensitivity, Specificity, Predictive Values, and Accuracy of Three Diagnostic Tests to Predict Inferior Alveolar Nerve Blockade Failure in Symptomatic Irreversible Pulpitis

**DOI:** 10.1155/2017/3108940

**Published:** 2017-06-14

**Authors:** Daniel Chavarría-Bolaños, Laura Rodríguez-Wong, Danny Noguera-González, Vicente Esparza-Villalpando, Mauricio Montero-Aguilar, Amaury Pozos-Guillén

**Affiliations:** ^1^Facultad de Odontología, Universidad de Costa Rica, San José, Costa Rica; ^2^Facultad de Odontología, Universidad Latina de Costa Rica, San José, Costa Rica; ^3^Doctorado Institucional en Ingeniería y Ciencia de Materiales, Universidad Autónoma de San Luis Potosí, San Luis Potosí, SLP, Mexico; ^4^Laboratorio de Ciencias Básicas, Facultad de Estomatología, Universidad Autónoma de San Luis Potosí, San Luis Potosí, SLP, Mexico

## Abstract

**Introduction:**

The inferior alveolar nerve block (IANB) is the most common anesthetic technique used on mandibular teeth during root canal treatment. Its success in the presence of preoperative inflammation is still controversial. The aim of this study was to evaluate the sensitivity, specificity, predictive values, and accuracy of three diagnostic tests used to predict IANB failure in symptomatic irreversible pulpitis (SIP).

**Methodology:**

A cross-sectional study was carried out on the mandibular molars of 53 patients with SIP. All patients received a single cartridge of mepivacaine 2% with 1 : 100000 epinephrine using the IANB technique. Three diagnostic clinical tests were performed to detect anesthetic failure. Anesthetic failure was defined as a positive painful response to any of the three tests. Sensitivity, specificity, predictive values, accuracy, and ROC curves were calculated and compared and significant differences were analyzed.

**Results:**

IANB failure was determined in 71.7% of the patients. The sensitivity scores for the three tests (lip numbness, the cold stimuli test, and responsiveness during endodontic access) were 0.03, 0.35, and 0.55, respectively, and the specificity score was determined as 1 for all of the tests. Clinically, none of the evaluated tests demonstrated a high enough accuracy (0.30, 0.53, and 0.68 for lip numbness, the cold stimuli test, and responsiveness during endodontic access, resp.). A comparison of the areas under the curve in the ROC analyses showed statistically significant differences between the three tests (*p* < 0.05).

**Conclusion:**

None of the analyzed tests demonstrated a high enough accuracy to be considered a reliable diagnostic tool for the prediction of anesthetic failure.

## 1. Introduction

The inferior alveolar nerve block (IANB) is the most common anesthetic technique used on mandibular teeth during root canal treatment [1–4]. Studies have reported a 30–80% failure rate for IANBs in patients with symptomatic irreversible pulpitis (SIP) [5–8]. Several theories have been proposed to explain local anesthetic failure; these theories include ionized anesthetic solutions in the inflamed tissue with a lower local pH, the overexpression and hyperactivity of voltage-gated sodium channels due to the presence of prostaglandins, and the overexpression of tetrodotoxin-resistant sodium channels [2, 5, 6, 8].

A successful IANB allows for comfort during the endodontic treatment. The diagnosis of anesthetic failure is challenging in endodontic clinical practice, especially in patients diagnosed with SIP [9, 10]. When this condition is misdiagnosed, pain can cause a patient to experience apprehension and anxiety during and after the endodontic treatment [11]. Some factors, such as lip numbness, a lack of response to cold stimuli, an electric pulp test, and an absence of pain during endodontic access, have been proposed to assess successful IANBs [6, 12, 13]. Several studies have reported that a negative response in the cold test or lip numbness does not guarantee pulp anesthesia [6, 8, 12–15]. Clinicians have been recommended to apply three diagnostic tests (lip numbness, lack of response to cold stimuli and absence of pain during endodontic access) to evaluate an IANB as a successful anesthetic technique [8].

Sensitivity and specificity determination studies have been used in the endodontic literature, mainly to confirm pulpal diagnostic tests [16–19]. This study employed a similar method to validate the diagnostic tests used to assess IANB failure. Therefore, the aim of the present study was to compare the sensitivity, specificity, and predictive values of lip numbness, the cold stimuli test, and responsiveness during endodontic access for IANB failure in the presence of SIP.

## 2. Materials and Methods

### 2.1. Study Design

This cross-sectional clinical study was conducted in accordance with the Declaration of Helsinki and was approved by the Institutional Ethics Committee of the Faculty of Dentistry according to STARD guidance for reporting diagnostic accuracy studies [20]. All subjects presented voluntarily to the Postgraduate Endodontics Clinic and were consecutively invited to participate in the study. The clinical procedures were explained, and written consent was obtained before any clinical test. All participants were informed that they could leave the study at any time before, during, or after the clinical procedures, with no consequences or loss of any privileges entitled to them before enrollment. The inclusion criteria to participate in the study were as follows: patients must be ≥18 years old; being diagnosed with SIP in the first or second mandibular molar with an indication for endodontic treatment; presenting acute pain categorized as moderate to severe according to a modified 100-mm Heft-Parker VAS [21], where the endpoints represented no pain and the worst experienced pain; and being without intake of analgesic or anti-inflammatory drugs for 12 hours prior to the treatment. The VAS was used during patient recruitment to homogenize the sample in order to avoid any bias concerning different levels of pain. The exclusion criteria were as follows: being pregnant, poor tooth integrity for restoration, crowned teeth, severe periodontal disease, radiographic signs of root resorption, dental fracture, noncontrolled systemic diseases such as diabetes or hypertension, intake of drugs or narcotics, sensory impairment or paresthesia in the orofacial region, or severe pain during the application of any test. If a patient decided not to continue his/her participation after any of the tests, the patient was immediately excluded, and no further tests were performed. These participants received additional anesthetic blockade using supplementary techniques until successful IANB was confirmed, and the root canal treatment was completed.

### 2.2. Clinical Procedures

The time for all clinical interventions was standardized by scheduling the procedures after 9:00 am and before midday. The IANB technique and the diagnostic procedures were conducted by a trained postgraduate endodontic resident at the Endodontics Clinic. The initial diagnosis was performed by a single experienced clinician (specialist in endodontics); thermal cold testing was performed by applying cold spray (Endo-Ice (1,1,1,2-tetrafluoroethane); Hygenic Corp, Akron, OH) on a sterile cotton pellet to the middle-third of the buccal surface of the tooth until the patient responded. The patients were asked to indicate the intensity and the duration of the sharp thermal sensation once identified. SIP was diagnosed if the duration and intensity of the response to the cold tests increased compared to the contralateral control tooth. The diagnosis was verified by the absence of radiographic evidence of periapical pathosis. Endodontic treatment was indicated for these patients, and confirmation of compliance with the inclusion criteria was assessed.

All participants received an IANB using 1.8 mL of mepivacaine 2% with 1 : 100000 epinephrine (Scandonest 2% Special, Septodont, Saint Maur des Fosses, France). The anesthetic was injected with a metal syringe and a 27-gauge, 1.25-inch needle (Monoject; Sherwood Services, Mansfield, MA). The same clinician (postgraduate endodontic resident) carried out all of the anesthetic blockades using a direct (Halsted) approach. After 15 min, a progressive diagnosis was performed using three diagnostic tests to identify IANB failure, and the results were compared to the gold-standard test. Participants were instructed to notify the clinician when they experienced any discomfort or a painful sensation during the performance of each test. Patients with uncertain responses were excluded from the study, in addition to those who asked to be excluded at any time.

### 2.3. Diagnostic Tests

Four consecutive clinical procedures were evaluated as diagnostic tests to determine IANB failure, including lip numbness, the cold stimuli test, responsiveness during endodontic access, and dental pulp manipulation (gold standard).

#### 2.3.1. Lip Numbness Test

Using sterile gauze, the contralateral midlip was stabilized to assess the numbness of the anesthetized midlip. The clinician used sterile dental tweezers and gently pinched the lip to assess the absence of sensitivity. The lip surface was pinched three times at equal points from the midline to the corresponding mouth commissure. Any positive response to these stimuli was assessed as IANB failure.

#### 2.3.2. Cold Stimuli Test

The tooth was completely isolated, and the clinician sprayed a cotton pellet with a cold solution (Endo-Ice F; Coltene Whaledent Inc., Cuyahoga Falls, OH, USA). The cold pellet was immediately placed on the middle-third of the buccal surface of the tooth's crown to evaluate sensitivity.

#### 2.3.3. Responsiveness during Endodontic Access

A painful response or discomfort during access through the enamel and dentin or during the removal of any restoration or carious tissue before gaining direct access to the pulp chamber was used as the third test to determine anesthetic failure. All access instances were performed using a high-speed handpiece with air/water coolant and a new #4 metallic round bur.

#### 2.3.4. Dental Pulp Manipulation

The gold-standard criterion for anesthetic failure was determined as discomfort during the removal of the pulpal tissue. The extent of pulp removal was standardized until a complete pulpotomy was performed using a sterile endodontic excavator (Code 33L, Hu-Friedy Co., Chicago, IL).

For all participants, the first appointment concluded with the removal of the pulpal tissue to relieve their symptoms, and a second appointment was planned for the conclusion of the endodontic treatment. If the participant reported any discomfort during a diagnostic test, then the anesthetic blockade was categorized as a failure according to that particular test. If pain was reported by a patient, then no further tests were performed. When the gold-standard test elicited a painful response, a second cartridge of mepivacaine was given as a repetition of the IANB or the intrapulpal technique, and a painless endodontic treatment was performed.

### 2.4. Sample Size

In diagnostic tests that yield dichotomized outcomes (positive or negative), accuracy is evaluated according to sensitivity and specificity. Subsequently, sensitivity and specificity determine the capability of a diagnostic test compared to the gold standard, and these factors are not influenced by the prior probability of disease (prevalence). Based on this principle, in this study, the sample size calculation was performed with a type I error of 0.05 (significance of 95%) and a statistical power of 80% using a general formula for sample size to compare two proportions of the diagnostic test [22]:

(1) n=Zα/22×P−1−P−+ZβP11−P1+P21−P22P1−P22,

where $\bar{P}$ is the average of *P*_1_ and *P*_2_, and *Z*_*α*_ and *Z*_*β*_ are the standard normal *Z* values that correspond to *α* and *β* (the probability of type I and type II errors, resp.). *P*_1_ was assumed to have a sensitivity value of 100% for the gold standard (dental pulp manipulation) to detect IANB failure, and *P*_2_ had a sensitivity value (84%) that corresponds to the cold test based on a previous report [23]. With the other diagnostic tests, we assumed lower sensitivity so that the sample size calculation was sufficient for the other diagnostic tests (lip numbness and responsiveness during endodontic access). Accordingly, 44 patients were required; however, 53 patients were included. This number of patients was considered acceptable to demonstrate the possible differences attributed to the diagnostic tests used.

### 2.5. Statistical Analysis

The number of true-positive (TP), false-positive (FP), true-negative (TN), and false-negative (FN) test results was calculated for each test conducted and was compared to the gold standard. According to these results, sensitivity (SN), specificity (SP), positive predictive value (PPV), negative predictive value (NPV), and accuracy (AC) with confidence intervals (CI 95%) were calculated for each test. Accuracy was calculated as the proportion of true results (both true positives and true negatives) among the total number of cases examined. In a confusion matrix, it is defined as follows:

(2) Accuracy=NTrue  Positives+NTrue  NegativesNTrue  Positives+NTrue  Negatives+NFalse  Positives+NFalse  Negatives.

For diagnostic precision, a Youden Index with a CI 95% was used. The index has a value of zero if the test reports the same proportion of positive tests for both the control group and the disease group. It has value unity when and only when neither false positives nor false negatives emerge from the test [24–27]. Finally, a receiver-operating characteristic (ROC) curve analysis was used to examine the overall discriminatory power and to calculate the area under the curve (AUC) for each test [28]. Comparisons of the ROC curves were conducted with a nonparametric approach using the theory of generalized* U*-statistics to generate an estimated covariance matrix (DeLong's test) and were considered significant at *p* < 0.05 [29]. Additionally, statistical analyses to associate variables (age, sex, and initial pain) with anesthetic failure were conducted (Fisher exact test).

## 3. Results

**Figure 1:**
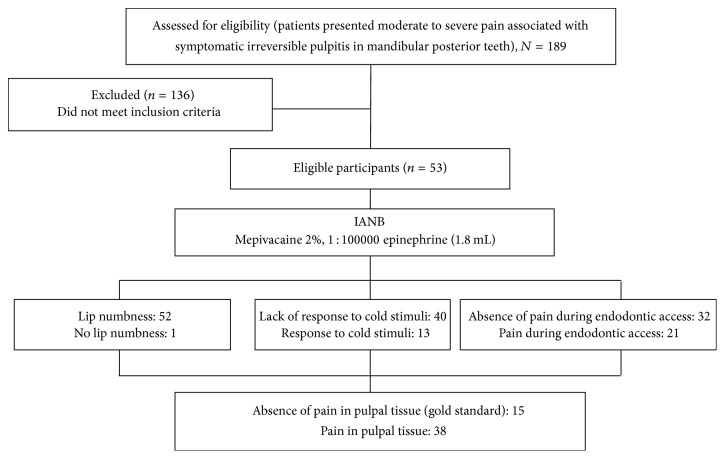
A flowchart of the clinical tests performed to predict anesthetic failure.

**Figure 2:**
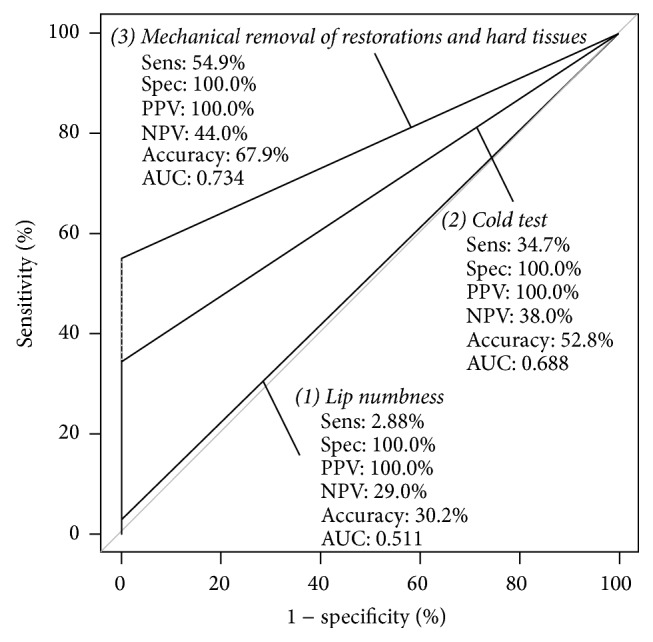
ROC curve analyses for the diagnostic tests.

Fifty-three patients (16 males and 37 females) were included in this study. The age range was 18–50 years, and the mean age was 33.75 ± 12.25 years. Overall IANB success was achieved in 15 subjects (28.3%), as determined by the gold-standard test.  Figure 1 presents a flowchart of the diagnostic tests performed and the clinical results obtained. As shown, all 53 eligible patients completed the diagnostic sequence and were included in the statistical analyses. No participants withdrew voluntarily or were excluded from the study, and no adverse events were observed during the diagnostic period. When analyzing the ability of the diagnostic tests to identify the 38 IANB failures, the lip numbness test identified one subject (TP), whereas 37 subjects reported lip numbness (FN). The cold stimuli test correctly identified 13 IANB failures (TP), and 25 subjects responded negatively (FN). Responsiveness during endodontic access identified 21 of the 38 IANB failures (TP), whereas 17 patients reported that they felt no pain during access (FN). The SN, SP, PPV, NPV, and AC values for each diagnostic test are presented in Table 1. The ROC curves results are graphically presented in Figure 2. All comparisons of the AUC scores between the three diagnostic tests showed significant differences (*p* < 0.05).

Additionally, no significant association was observed between IANB success and age (*p* = 0.122), sex (*p* = 0.754), or initial pain (*p* = 0.131).

## 4. Discussion

This study compared the accuracy of lip numbness, the cold stimuli test, and responsiveness during endodontic access as diagnostic tests for the detection of IANB failure. No diagnostic test appeared to be a reliable approach to determine the effectiveness of this anesthetic technique in patients diagnosed with SIP. Patients with SIP are known to experience anesthetic failure in mandibular molars, a situation that is extensively recognized in previous reports [5, 30]. Several clinical and pharmacological strategies such as supplementary techniques or analgesic premedication are recommended to prevent painful treatments and patient discomfort. Thorough anesthesia throughout the entire endodontic treatment promotes a more comfortable postoperative recovery and can decrease analgesic drug consumption [8, 31, 32].

Regarding the ethical aspects of the study, since an anesthetic blockade was performed, the tests themselves are not pain inflictors, but clinical procedures were selected to identify anesthetic failure. All participants consented to participation and were fully aware that potential discomfort may present during the tests. This discomfort is a reasonable response to anesthetic failure. A positive response to any of the tests was classified as anesthetic failure according to that test. The response to anesthetic failure varies from person to person, ranging from light sensation to discomfort. Independent of responsiveness and in order to avoid subjectivity in the study, neither the duration nor the intensity of the response was measured, and only positive or negative reactions were taken into account. When the response was considered as severe pain, the patient could have been excluded, and no further tests would have been performed. Moreover, during the study, no participants asked to be excluded, and all endodontic procedures were routinely completed.

In a clinical study of 61 patients, 100% reported lip numbness, but only 62% reported pulp anesthesia using the electric test. Another study stated that 84% of 256 endodontists reported that when the IANB technique was used in the presence of SIP, although the patients reported lip numbness, they experienced pain during endodontic access [33]. Clinical studies reported a 30%–80% probability of failure when IANB was used in molars of patients with SIP [2, 4, 33, 34]. The present study confirmed the high prevalence of IANB failure (71.7%) among patients with SIP. The clinical relevance of this finding is determined by the ability of the clinician to detect IANB failure before gaining access to the dental pulp and causing unnecessary pain to the patient.

Traditionally, dentists have confirmed anesthetic success through subjective methods, such as asking the patients about their symptoms or soft tissue testing. More recently, the use of thermal or electrical stimuli to assess pulpal anesthesia as more objective tests has been proposed [6, 8, 12, 13, 35–37]. Therefore, the ability of these tests to determine anesthetic failure has not been reported. For this study, the direct manipulation of the dental pulp was the gold-standard procedure for anesthetic success, as previously reported [8, 13]. This selected diagnostic sequence is applicable only for the endodontic management of teeth using the SIP model. The generalizability of the results obtained and the gold standard used in the present study are limited to IANB failure in SIP (and not to more sophisticated surgical setups). In this model, dental pulp manipulation, which is clinically the deepest tissue to be manipulated, represents direct physical contact with the nerve endings. The results of the present study showed that, every time an IANB was successfully achieved (*n* = 15), the participants responded negatively in all three tests, accurately identifying the success (SP = 1.00), and no false negatives were obtained when performing these tests. Based on the perfect specificity of the results, ROC curves for all three tests were drawn as straight lines and not as curves [28]. Additionally, the study found a 100% probability that when subjects had a positive result in any of the three tests, they truly had IANB failure. Regarding the predictive value calculations, the PPV was defined as the percentage of subjects with a positive test result (lip sensitivity, responsiveness to cold stimuli, or symptomatic pulp access) who truly had IANB failure (condition), and the NPV was defined as the percentage of subjects with a negative test result (lip numbness, no response to cold stimuli, or asymptomatic pulp access) who truly had a successful IANB (lack of condition). Our results showed that the PPV values were equal to 1 for all three tests, but the NPV values increased in the order of lip numbness, the cold stimuli test, and responsiveness during endodontic access (values of 0.29, 0.38, and 0.44, resp.). Clinically, the PPV indicates that any time that a test was positive, IANB failure can be expected. Moreover, for the NPV in 71% of patients with lip numbness, failure may be present. The same outcome could occur in 62% of patients with a negative cold test result and 56% of patients with no responsiveness during endodontic access.

When comparing the accuracy of the three tests to correctly identify IANB failure, the responsiveness during endodontic access (SN = 0.55) showed the best scores; however, it identified only one of every two failures. When analyzing the ROC curves and the AUC scores (0.73) of these tests, responsiveness during endodontic access again showed the best accuracy, but it can only be classified as a fair accuracy test. The cold stimuli test has been shown to be modest when screening for IANB failure (SN = 0.35). Randomized clinical trials have reported that patients with mandibular molars with SIP and negative responses to cold tests may still report moderate to severe pain during endodontic access [8, 13]. Accordingly, this study showed that when subjects reported a negative response to cold stimuli, only 38% of the IANBs were truly successful. The literature suggests that lip numbness is a poor test when screening for IANB failure and should not be used clinically [13, 35–37]. The present study supports the evidence against the use of this test to assess IANB failure. When viewing the ROC curve for lip numbness, the AUC score of 0.51 indicates that it is almost useless for identifying IANB failure and that the results obtained from conducting this test could be due to chance.

When evaluating anesthetic failure, the most conclusive evidence of the technique's performance would be an assessment of discomfort or pain during pulp manipulation [8, 13, 30]. Consequently, this study used this measure as the gold-standard test for comparison with the other tests. The application of a single diagnostic test to determine the anesthetic status of inflamed pulpal tissue seems to be insufficient; therefore, future investigations should look for tests that are more accurate or a combination of tests to predict this failure. Little evidence is available to completely explain the different validities of the three tests. However, several biological explanations can contribute to the understanding of this phenomenon. The first test, lip numbness, evaluates the mechanical response to proprioception. Therefore, the clinician could only assume IANB success after inhibiting the pressure sensitization. For this reason, lip numbness does not necessarily indicate pulpal anesthesia [30]. The cold stimuli test, though it represents a standard well-known strategy to identify pulp sensitivity [38], relies on the transmission of the temperature from a cotton pellet to the dental pulp. The final temperature that reaches the pulp tissue depends not only on the stabilization of the temperature from the external source to the oral environment but also on its transmission through the enamel and dentin tissues [39]. Additionally, the thermal response of partially anesthetized pulp can elicit a negative response on the peripheral nerve endings of the pulp (A-*δ* fibers), but the inner pulp areas, which are rich in C-fiber nerve endings, may not be stimulated by cold and can be underdiagnosed [40, 41]. A similar situation could apply to responsiveness during the endodontic access test, where the painless removal of peripheral dental structures could mask a failed IANB due to an incomplete anesthetic effect. Preoperative inflammation of the dental pulp, especially deep pulpal tissue, could explain this behavior based on a lower local pH, TTX-r sodium channel overexpression, increased prostaglandins E2 levels, and increased vasodilation [5, 13].

A limitation to the design of this study was the short period of time between recruitment, diagnosis, and treatment. Even when this situation is desirable under the terms of this model, it did not allow for the exploration of the psychological or emotional features of the participants that were already suffering from dental pain. For this reason, the sample could have been skewed towards more emotional subjects. The diagnostic tests performed were part of a routine clinical procedure that may inevitably elicit some level of pain due to IANB failure in some cases, which could have biased the sampling. However, this was not considered a limitation because all participants completed the experiment, and no participation refusal was recorded. Another limitation that will be taken into account in further research is the lack of a psychological evaluation during the recruitment interview. Although all participants were strictly selected and guided to homogenize the final sample, individual perceptions of pain and the clinical procedure may have influenced the responses. Pain catastrophizing may be associated with the negative appraisal of pain-related threats [42] and may affect the outcomes of research according to the pain intensity level [43]. Dental patients would not only passively receive a painful stimulus but also actively associate their experienced pain with the context in which they received the stimulus [44]. Considering the psychological characteristics of the participants is important due to the subjective nature of pain and the diagnostic sequence followed by the researchers. Since all three tests had to be conducted consecutively in a relatively short period, a placebo effect or exaggerated unpleasantness in painful situations and expectations of negative outcomes (referring to the magnification element within the pain catastrophizing phenomenon) could have been present in the patients [42], especially since they were all suffering from pain, and they had already received the anesthetic injection. Other limitations of the study were the impossibility of achieving blindness to the diagnostic sequence and the lack of interobserver agreement calculation. The impact of these situations must be evaluated in future studies.

Validation methods for diagnostic tests are becoming a trend [45, 46]. In general, the accuracy estimations obtained in the diagnostic tests are overoptimistic [47]. As alternatives to address this situation, validation methods such as real cross-validation, split-half cross-validation, and bootstrapped cross-validation are available. These techniques tend to avoid the use of an entire dataset when accuracy estimates are obtained [48]. With these validation strategies, the diagnostic tests have advantages to address overoptimistic estimations and confidence parameters and to address small sample sizes [47, 49].

Finally, when treating a patient with SIP, clinicians must cautiously evaluate the success of the IANB before manipulating the pulp tissue and consider that failure of the anesthetic technique, and consequently pain, may present at different stages of the clinical procedure.

## 5. Conclusions

Anesthetic failure is a common and challenging condition to diagnose when using the IANB technique in patients with SIP. IANB failure was determined in 71.7% of the patients according to the gold-standard test. The test that showed the best validity was responsiveness during endodontic access, with SN, SP, PPV, and NPV values of 0.55, 1.00, 1.00, and 0.44, respectively. Clinically, none of the evaluated tests demonstrated a high enough accuracy to be considered a reliable diagnostic tool for this condition (values of 0.30, 0.53, and 0.68 for lip numbness, the cold stimuli test, and responsiveness during endodontic access, resp.).

## Figures and Tables

**Table 1:** Sensitivity, specificity, predictive values (positive (PPV) and negative (NPV)), accuracy, the Youden Index, and the statistical power for the diagnostic tests.

	Sensitivity (CI 95%)	Specificity (CI 95%)	PPV (CI 95%)	NPV (CI 95%)	Accuracy (CI 95%)	Youden Index (CI 95%)	Statistical power
Lip numbness	0.03 (0.00, 0.13)	1.00 (0.69, 1)	1.00 (0.01, 1)	0.29 (0.17, 0.43)	0.30 (0.18, 0.44)	0.03 (−0.3, 0.1)	0.0212
Cold stimuli test	0.35 (0.2, 0.51)	1.00 (0.7, 1)	1.00 (0.66, 1)	0.38 (0.22, 0.54)	0.53 (0.39, 0.67)	0.34 (−0.1, 0.51)	0.9625
Responsiveness during endodontic access	0.55 (0.37, 0.7)	1.00 (0.68, 1)	1.00 (0.77, 1)	0.44 (0.26, 0.62)	0.68 (0.54, 0.80)	0.54 (0.05, 0.7)	0.9999
